# Circulating Carboxylated Osteocalcin Correlates With Skeletal Muscle Mass and Risk of Fall in Postmenopausal Osteoporotic Women

**DOI:** 10.3389/fendo.2021.669704

**Published:** 2021-05-05

**Authors:** Jacopo Antonino Vitale, Veronica Sansoni, Martina Faraldi, Carmelo Messina, Chiara Verdelli, Giovanni Lombardi, Sabrina Corbetta

**Affiliations:** ^1^ Laboratory of Movement and Sport Science, IRCCS Istituto Ortopedico Galeazzi, Milan, Italy; ^2^ Laboratory of Experimental Biochemistry and Molecular Biology, IRCCS Istituto Ortopedico Galeazzi, Milan, Italy; ^3^ Radiology Unit, IRCCS Istituto Ortopedico Galeazzi, Milan, Italy; ^4^ Department of Biomedical Sciences for Health, University of Milan, Milan, Italy; ^5^ Laboratory of Experimental Endocrinology, IRCCS Istituto Ortopedico Galeazzi, Milan, Italy; ^6^ Department of Athletics, Strength and Conditioning, Poznań University of Physical Education, Poznań, Poland; ^7^ Endocrinology and Diabetology Service, IRCCS Istituto Ortopedico Galeazzi, Milan, Italy; ^8^ Department of Biomedical, Surgical and Dental Sciences, University of Milan, Milan, Italy

**Keywords:** osteocalcin, skeletal muscle mass, risk of fall, osteoporosis, fragility fractures

## Abstract

**Background:**

Bone and skeletal muscle represent a single functional unit. We cross-sectionally investigated body composition, risk of fall and circulating osteocalcin (OC) isoforms in osteoporotic postmenopausal women to test the hypothesis of an involvement of OC in the bone-muscle crosstalk.

**Materials and Methods:**

Twenty-nine non-diabetic, non-obese, postmenopausal osteoporotic women (age 72.4 ± 6.8 years; BMI 23.0 ± 3.3 kg/m^2^) underwent to: 1) fasting blood sampling for biochemical and hormone assays, including carboxylated (cOC) and uncarboxylated (uOC) osteocalcin; 2) whole-body dual energy X-ray absorptiometry (DXA) to assess total and regional body composition; 3) magnetic resonance imaging to determine cross-sectional muscle area (CSA) and intermuscular adipose tissue (IMAT) of thigh muscles; 4) risk of fall assessment through the OAK system.

**Results:**

Appendicular skeletal muscle index (ASMMI) was low in 45% of patients. Forty percent got a low OAK score, consistent with moderate-severe risk of fall, which was predicted by low legs lean mass and increased total fat mass. Circulating cOC levels showed significantly correlated with βCTx-I, lean mass parameters including IMAT, and OAK score. Fractured and unfractured women did not differ for any of the analyzed parameters, though cOC and uOC positively correlated with legs lean mass, OAK score and bone markers only in fractured women.

**Conclusions:**

Data supported the relationship between OC and skeletal muscle mass and function in postmenopausal osteoporotic women. Serum cOC, but not uOC, emerges as mediator in the bone-muscle crosstalk. Circulating cOC and uOC levels may be differentially regulated in fractured and unfractured osteoporotic women, suggesting underlying differences in bone metabolism.

## Introduction

Aging results in the progressive and parallel loss of bone, known as osteopenia, and in skeletal muscles, known as sarcopenia. Sarcopenia is defined as the loss of skeletal muscle mass and quality, which accelerates with aging and is associated with functional decline. Osteopenia and sarcopenia are two main determinants of aging-related fragility (1), and sarcopenia represents one of the main causes of increased risk of falls and, directly or indirectly, fractures (2). Sarcopenia in elderly women associated with an increased risk of all-cause mortality (3, 4). Moreover, in older adults, the coexistence of osteopenia and sarcopenia, namely osteosarcopenia, has to be regarded as the major risk factor for fractures and further functional decline due to low physical performance (5–7). Among the lean tissues, skeletal muscles exert a strong positive effect on bone mass (8–10), while the impact of fat is weaker and likely indirect (11, 12).

Bone and muscle are functionally related, not only biomechanically since the direct connection, but also based on the emerging intense endocrine crosstalk (13–16).

Osteocalcin (OC) is mainly secreted by osteoblasts during bone formation, in part also by osteocytes, and it binds to the mineralized matrix (17). Its role in skeletal remodeling is debated as OC knockout mice showed normal bone mineral density though they display a crystals misalignment along the collagen fibrils consistent with a low degree of crystal maturation and increased brittleness (18, 19). OC overexpression in mice does not affect bone mineralization, but it promotes recruitment and differentiation of circulating monocytes and osteoclast precursors, suggesting a role in the osteoblast-osteoclast interaction (20).

Among other metabolic abnormalities and the substantially unmodified bony phenotype, mice in which Gprc6a, the putative receptor for OC, has been knocked out experienced decreased muscle mass, while the knockout mice of Esp, a phosphatase that inhibits the function of OC, has increased muscle mass. Further evidence that OC may solve a relevant role in muscle mass gain and muscle function is that supplementation with OC restores reduced exercise capacity in aged mice and increases muscle strength. Aerobic exercise increases circulating bioactive OC levels (i.e., uncarboxylated OC) and induces OC signaling in muscle leading to the expression of the myokine IL-6 (21, 22).

OC regulates muscle mass independently of its effects on energy expenditure, acting by direct activation of the receptor GPRC6A (21–23). Uncarboxylated OC (uOC) seems to directly promote protein synthesis in mice myotubes, explaining why this hormone is responsible for muscle maintenance during aging (21, 22). Moreover, uOC induces myoblast proliferation *via* sequential activation of the PI3K/Akt and p38 MAPK pathways in C2C12 murine myoblasts, while it enhances myogenic differentiation *via* a mechanism involving GPRC6A-ERK1/2 signaling (23).

However, many studies were published using animal models, and they need to be confirmed in humans. Some differences between murine and human OC should be considered. OC is carboxylated on glutamic acid residues (Glu→Gla) 13, 17, and 20 in the mouse protein and on Glu 17, 21, and 24 in humans. Moreover, regarding the circadian rhythm, in mice OC levels peak during the daytime and are at lowest during nighttime, whereas in humans, the levels fall in the early morning, rise in the afternoon and peak at night (14, 24).

The primary aim of the present study was to investigate the associations between circulating carboxylated (cOC) and uOC, body composition (i.e., bone, fat and muscle mass) and risk of fall in a series of postmenopausal osteoporotic elderly women. Secondly, we aimed to examine the pairwise differences in body composition, risk of fall and circulating cOC and uOC levels between fractured and unfractured osteoporotic women. We tested the hypothesis that cOC and/or uOC are involved in the bone-muscle crosstalk in osteoporotic elderly women.

## Materials and Methods

### Study Design

This is an observational cross-sectional study conducted in accordance with the STROBE guidelines for cross-sectional studies (25) and was approved by the Ethical Committee of Vita-Salute San Raffaele University (ref.no.17/INT/2017). Before the beginning of the study, all the participants signed their written informed consent to participate. All study procedures were performed in compliance with the laws and regulations governing the use of human subjects (Declaration of Helsinki) and the study protocol was registered at clinicaltrials.gov (ref.no. NCT03382366). All patients enrolled were investigated by: a) clinical and anthropometric evaluation; b) risk of fall evaluation by the OAK system (Khymeia, Noventa Padovana, Italy); c) fasting blood sample for biochemical and hormonal assays, and d) total body dual energy x-ray absorptiometry scan (DXA) for body composition assessment. Moreover, magnetic resonance imaging (MRI) was performed to measure the cross-sectional muscle area (CSA) and the intermuscular adipose tissue (IMAT) of thigh muscles.

### Study Population

**Figure 1:**
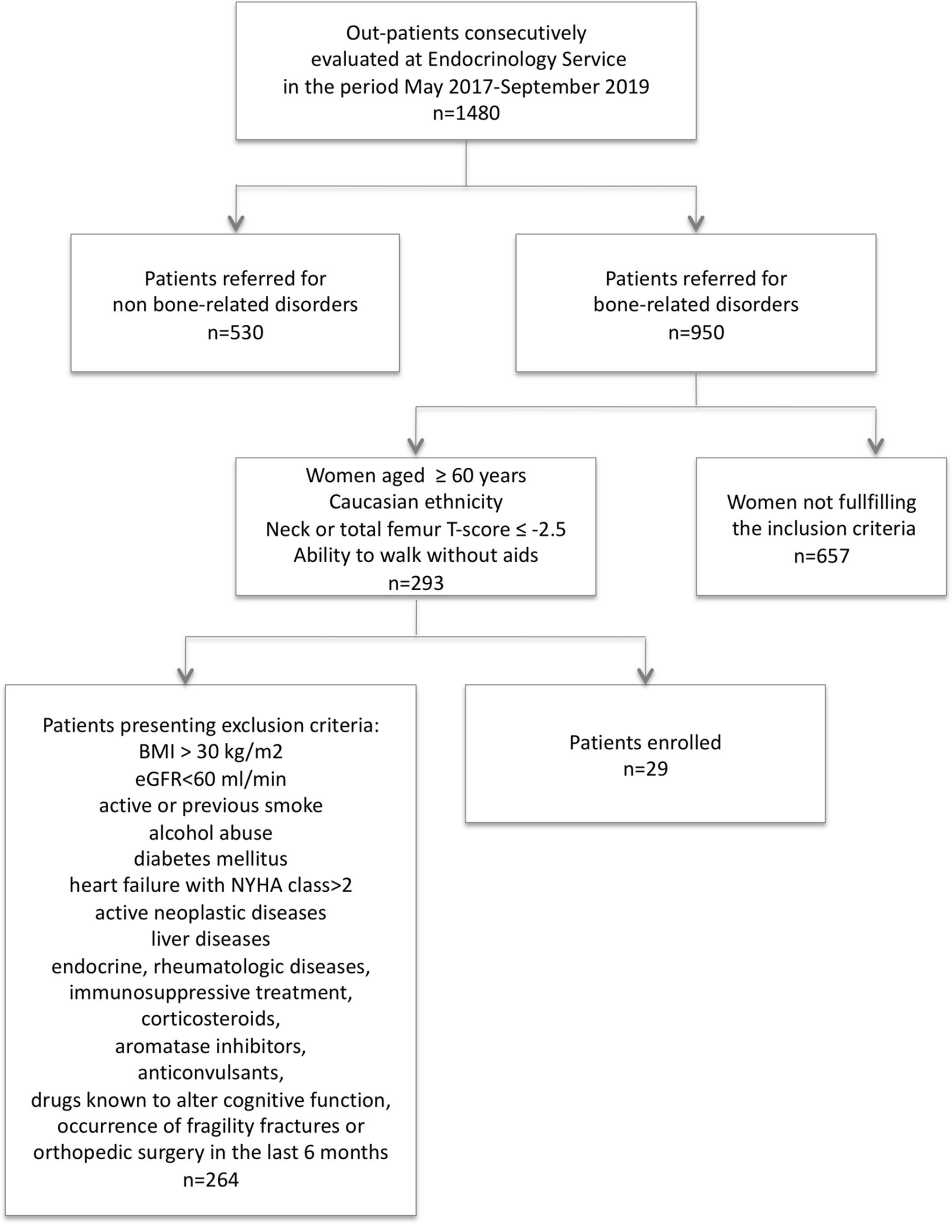
Diagram representing process of patients enrollment.

The final series was represented by 29 postmenopausal non-obese women (mean age 72.4±6.8, range 60-85 years; BMI 23.0±3.3 kg/m^2^, range 18.1-29.3) from the outpatients referred to the Endocrinology Service of IRCCS Istituto Ortopedico Galeazzi in Milan. Women were consecutively enrolled between May 2017 and September 2019 as illustrated in **Figure 1**. Inclusion criteria were: Caucasian ethnicity, age ≥ 60 years, a DXA-based diagnosis of osteoporosis at proximal hip (neck or total femur bone mineral density (BMD) T-score ≤ -2.5), and ability to walk without aids. Exclusion criteria included: age< 60 years, BMI> 30 kg/m^2^, estimated GFR> 60 ml/min, active or previous smoke, alcohol abuse, diabetes mellitus, heart failure with NYHA class> 2, active neoplastic diseases, liver diseases, ascertained endocrine and rheumatologic diseases, immunosuppressive treatment including corticosteroids, treatment with aromatase inhibitors, anticonvulsants, drugs known to alter cognitive function, occurrence of fragility fractures or orthopedic surgery in the last 6 months before the enrollment. All women, included those with previous fragility fractures, were free from anti-osteoporotic drugs, calcium and vitamin D supplementation since at least 6 months.

### Fall Risk Assessment by OAK Device

The OAK device is a safe and validated device used for the assessment of fall risk [26]; it provides an automated version of the Brief-BESTest, a clinical tool examining balance performance in six specific context of postural control (26). The risk of fall reflects bone frailty, chronic, and/or degenerative conditions associated with physical, sensory, and cognitive changes in advancing age (27). The OAK system works semi-automatically and an experienced investigator assisted every session. The OAK system comprises two stabilometric platforms, three sensorized bars, four antennas and a Human Machine Interface providing audio and video instructions to the subjects. Before the beginning of the test, all subjects wore a portable device connected to a set of four inertial-magnetic sensors, located on the wrists and the thighs through gloves and rip-ties, to interact with the OAK system. Data from sensors, platforms and bars were then collected and integrated to calculate balance scores for each task. The global score ranges between 0 and 24 points: a score between 17 and 24 classifies a subject as low risk of fall, while a score between 0 and 16 identifies a subject as medium/high risk of fall (28). A familiarization session was performed before the text execution; the time range needed to complete the whole test was 9-15 minutes.

### Body Composition Analysis

A whole-body DXA scan was performed to measure total and regional body composition (Hologic QDR-Discovery 139 W densitometer; Hologic Inc., Bedford, MA, USA). Regions of interest were automatically defined by the software including six different body districts: total body, trunk, upper limbs (left and right arms), lower limbs (left and right legs). For each region, the exam provided the weight of total mass, fat mass, and lean mass, all expressed in grams (g), as well as regional BMD values, expressed in g/cm^2^. The precision of the BMD measurement by DXA has coefficients of variation (CV) at different sites between 0.4% (lumbar spine) to 1.3% (femoral neck) and 2% to 6% for body composition, in line with previous report (29).

The total amount of lean mass was also investigated by using the Appendicular Skeletal Muscle Mass Index (ASMMI), which is the amount of muscle in the upper and lower limbs, corrected by the individuals’ square of the height (appendicular lean mass/height^2^). The most valid and widely accepted cut-off value for ASMMI with the whole-body DXA in women is reported to be 5.7 kg/m^2^, with subjects under this threshold being diagnosed with low muscle mass (30, 31). Diagnosis of osteoporosis was established in presence of BMD of −2.5 standard deviation or more below the mean of a young healthy adult (T-score) (32). DXA-measured femoral neck BMD (33) and ASMMI (31, 33) are considered the gold standard values for the diagnosis of osteoporosis and low muscle mass, respectively (34, 35).

### Magnetic Resonance Imaging (MRI)

MRI was performed in twenty-four women; the remaining patients refused to undergo this additional procedure due to claustrophobia. All scans were performed with a 1.5T MR system (Avanto, Siemens Medical Solution, Erlangen, Germany) and 15 slices with a thickness of 5 mm were acquired covering a total length of 7.5 cm at the middle third of the right thigh. MRI protocol included a transverse T1-weighted sequence (for anatomic reference) and a transverse Dixon sequence (for quantitative analysis), for a total examination time of about 10 minutes. More in detail, intermuscular adipose tissue (IMAT) quantification was performed by using Dixon MRI sequences, which produce four sets of MRI images providing information on water and fat content separately, therefore offering the possibility for precise fat quantification (36, 37). The segmentation of the thigh muscles was performed for each slice of that in which the muscle-tendon junction of the gluteus maximus muscle was visible with the use of ImageJ, an open-source software (38), by a single expert operator (C.M.). The whole muscle area was selected as a single unit. Two quantitative parameters were finally calculated using Image J15: the thigh cross-sectional muscle area (CSA), expressed in mm^2^, and the thigh IMAT, representing the IMAT absolute value of the total muscle CSA and expressed in mm^2^. Subcutaneous fat, major blood vessels and the bony femur were excluded from the segmentation.

### Laboratory Examination

Fasting blood samples were collected from each patient by standard venipuncture. Total calcium, phosphate, total alkaline phosphatase activity (ALP), creatinine, and the bone resorption marker type I collagen C-terminal cross-linked telopeptide (βCTx-I) were measured by routine assays in serum tubes with clot activator (SSTII Advance Vacutainer, Becton Dickson, Franklin Lakes, NJ, USA). Dipotassium ethylendiaminotetraacetate (K2EDTA)-anticoagulated plasma PTH (K2EDTA Vacutainer, Becton Dickinson) and serum 25-hydroxyvitamin D [25-(OH)D)] were assayed by Roche. Plasma cOC and uOC were measured by the means of two specific monoclonal antibody-based sandwich immunoassays (Undercarboxylated OC EIA kit and Gla-Type OC EIA kit, Takara Bio Inc., Otsu-Shi, SHG, Japan). The lower limit of detection (LLD) was 0.25 ng/mL for both assays. As reported by the manufacturer, intra-assay (CV_w_) and interassay (CV_b_) coefficients of variation were 4.58% and 5.67% for uOC and 3.3% and 1.0% for cOC, respectively (39). All samples were tested in duplicate and according to the most up-to-date pre-analytical warnings (40, 41).

### Statistical Analysis

Sample size was calculated by G*power3.1, considering as significant a correlation with a slope of at least 0.48, an α-error of 0.05 and a power of 0.80 in a model of linear bivariate regression. Continuous variables were given as mean ± standard error media (SEM). Numeration data were described as percentages (%). The normality of the distribution of clinical, radiological and laboratory variables for the fractured (n=13) and unfractured group (n=16) were checked using graphical methods and the Shapiro-Wilk test. Data homogeneity between groups was tested through un-paired Student t-tests or with the Mann-Whitney rank test for non-normally distributed variables. Significance was set at p<0.05. In addition, the normality of the distribution of clinical, radiological and laboratory variables were checked using graphical methods and the Shapiro-Wilk test for the entire group of subjects. The existence of a correlation between outcomes was tested by the Pearson’s correlation index. The same approach was adopted to test the existence of possible correlations between cOC or uOC and the other outcomes for the group of fractured and unfractured women separately. Correlations were considered significant when r>0.25 and P<0.05. Multivariate analysis considering OAK score and cOC as dependent variables have been performed to test the hypothesis they could be predictive of muscle parameters. Statistical analysis was performed using GraphPad Prism version 6.00 (GraphPad Software, San Diego, CA, USA) and by Past3.14 (42).

## Results

### Body Composition in the Series of Postmenopausal Osteoporotic Women

**Figure 2:**
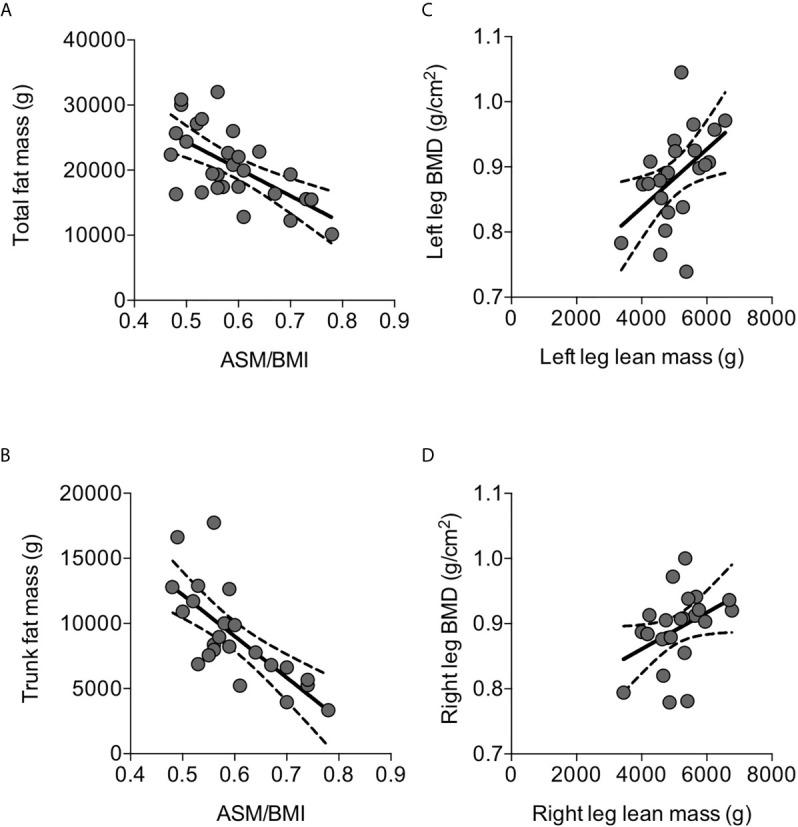
Correlations between parameters of body composition detected by DXA in osteoporotic postmenopausal women. ASM/BMI negatively correlated with total fat mass **(A)** and, in particular, with trunk fat mass **(B)**. Left leg lean mass positively correlated with the corresponding segmental BMD **(C)**; similar correlation was detected between the right lean mass and the corresponding segmental BMD **(D)**. Lines represent regressions, dot lines represent 95% confidence intervals. Data were analyzed by Pearson coefficient of correlation.

Mean ASMMI was 5.69±0.13 kg/m^2^. Considering a cut-off of 5.7 kg/m^2^ for the diagnosis of low muscle mass in elderly women according the Consensus Report produced by European Working Group on Sarcopenia in Older People 2 (EWGSOP2) (30), low muscle mass was detected in 13 (45%) out of the 29 osteoporotic women, with ASMMI ranging between 4.20 and 5.62 kg/m^2^. In the present series of osteoporotic postmenopausal women, ASMMI did not correlate with age, while it positively correlated with BMI value (r=0.599, p=0.006); therefore, the muscle mass index was normalized by BMI, and appendicular skeletal muscle (ASM)/BMI was considered. ASM/BMI negatively correlated with total fat mass (r=-0.644, p=0.0002) (**Figure 2A**) and in particular with the trunk fat mass (r=-0.724, p=0.0001) (**Figure 2B**). Despite ASM/BMI did not show any significant correlation with segmental bone mineral density, considering the lean mass of each leg, a significant positive correlation with the corresponding segmental bone mineral density (BMD) emerged (**Figures 2C, D**).

**Figure 3:**
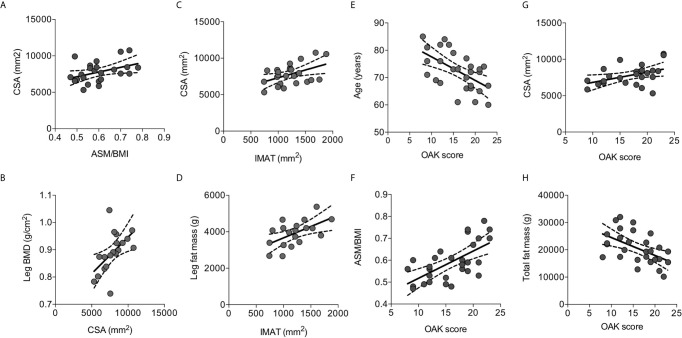
Correlations between parameters of body composition detected by MRI and with risk of fall measured by OAK automated system. Thigh cross sectional area (CSA) positively correlated with ASM/BMI **(A)** and with the leg BMD **(B)**. The intramuscular adipose tissue (IMAT) positively correlated with CSA **(C)** and with the leg fat mass **(D)**. OAK scores negatively correlated with age **(E)**, positively with ASM/BMI **(F)** and with CSA **(G)**, and negatively with total fat mass **(H)**. Lines represent regressions, dot lines represent 95% confidence intervals. Data were analyzed by Pearson coefficient of correlation.

We further gained insight about skeletal muscle mass features in elderly osteoporotic women investigating the muscle and fat components of thigh by MRI. The thigh CSA ranged 5323- 10759 mm^2^, and positively correlated with ASM/BMI (r=0.415, p=0.044) (**Figure 3A**) and with leg BMD (r=0.554, p=0.014) (**Figure 3B**).

The extracellular adipose tissue found beneath the fascia and in-between muscle groups, evaluated as IMAT, ranged between 741 mm^2^ and 1881 mm^2^, representing 10-25% of the thigh CSA. IMAT positively correlated with CSA (r=0.471, p=0.020) (**Figure 3C**), and with the leg fat mass measured by DXA (r=0.564, p=0.012) (**Figure 3D**).

Fall risk was evaluated using the OAK score derived by the OAK system. Mean OAK score was 17.8 ± 4.7 point-score out of 24.0; 12 women (41%) showed an OAK score between 0-16 consistent with moderate-high risk of fall. OAK scores negatively correlated with age (**Figure 3E**). Interestingly, OAK scores positively correlated with ASM/BMI (r=0.645, p=0.0002) (**Figure 3F**), with CSA (r=0.436, p=0.033) (**Figure 3G**), and negatively with total fat mass (r=0.527, P=0.003) (**Figure 3H**). A multivariate model considering OAK score as the dependent variable, and age, ASM/BMI, CSA, and total fat mass as the independent variables, indicated that OAK score is significantly related to age (r^2^ = 0.334, p=0.004) as well as to ASM/BMI (r^2^ = 0.416, p=0.005).

### Circulating Osteocalcin Levels in Osteoporotic Postmenopausal Women

OC is released by activated osteoblasts (17). Though both plasma cOC and uOC did not show any significant correlation with segmental BMDs nor with total or segmental fat masses (data not shown), in the series of osteoporotic women, both serum cOC and uOC levels positively correlated with serum βCTx-I levels, while any significant correlation could be detected with the mineral metabolic markers (**Table 1**). It is note of worth that plasma cOC, but not uOC, levels positively correlated with the ASM/BMI (**Table 1**). Considering the different skeletal muscle segments, plasma cOC, but not uOC, levels positively correlated with trunk lean mass as well as with legs lean mass (**Table 1**). In line with finding in DXA-derived lean mass parameters, plasma cOC levels positively correlated with CSA and with IMAT measured by MRI (**Table 1**). Interestingly, plasma cOC levels also positively correlated with muscle function assessed as OAK score (**Table 1**). In a multivariate model considering plasma cOC levels as the dependent variable, and serum βCTx-I levels, legs lean mass, IMAT, and OAK score, as the independent variables, cOC levels were significantly related to serum βCTx-I levels (r^2^ = 0.147, p=0.011) and to legs lean mass (r^2^ = 0.319, p=0.017).

**Table 1:** Significant correlations between plasma cOC and uOC levels and parameters related to bone, muscle mass, and muscle function.

Pearson	cOC		uOC	
	r	P	r	P
***Bone parameters***				
βCTx-I	0.620	**0.039**	0.707	**<0.0001**
***Muscle mass***				
ASM/BMI	0.458	**0.013**	0.009	0.961
Trunk lean mass	0.458	**0.012**	0.186	0.400
Legs lean mass	0.565	**0.001**	-0.134	0.542
CSA	0.519	**0.009**	0.153	0.475
IMAT	0.435	**0.034**	-0.022	0.917
***Muscle function***				
OAK score	0.431	**0.020**	0.014	0.944

cOC, carboxylated osteocalcin; uOC, uncarboxylated osteocalcin; r, coefficient of correlation by Pearson test; P, statistical significance; βCTx-I, beta-crosslinked C-terminal telopeptide of type I collagen; ASM/BMI, appendicular skeletal muscle mass corrected for BMI; CSA, Cross-sectional muscle area of the thigh; IMAT, Intermuscular fat of the thigh. Bold values highlighted statistical significant values.

### Differences Between Fractured and Unfractured Osteoporotic Women

Thirteen women (45%) experienced previous fragility fractures: at least one vertebral fracture (clinical and morphometric vertebral fractures, detected by dorsal and lumbar conventional x-ray imaging) was diagnosed in 10 women, femur neck fracture in 1 woman, and distal radius fractures in 2 women. Fractured osteoporotic women did not show evident differences in their body composition when compared with unfractured women (**Table 2**).

**Table 2:** Pairwise comparisons between fractured (n=13) and unfractured (n=16) osteoporotic women for clinical, body composition and bone metabolism variables.

Parameters	Fractured women (n=13)	Unfractured women (n=16)	Significance
Age (years)	74.7±1.6	70.6±1.8	0.107
***Bone mineral density***			
L1-L4 T-score	-2.70±0.31	-3.00±0.26	0.448
Neck T-score	-2.74±0.09	-2.60±0.13	0.410
Femur T-score	-2.34±0.19	-2.51±0.15	0.487
Arm sBMD (g/cm^2^)	1.194±0.201	1.199±0.080	0.926
Legs BMD (g/cm^2^)	1.709±0.310	1.630±0.509	0.656
***Fat mass***			
BMI (kg/m^2^)	24.2±0.9	22.1±0.8	0.075
Total fat mass (g)	22109.0±1817.0	19283.0±1187.0	0.190
Arms fat mass (g)	3107.0±1272.0	2567.0±1242.2	0.316
Legs fat mass (g)	7735.0±1699.0	7889.0±1507.4	0.821
Trunk fat (g)	9988.0±1307.0	8163.0±872.0	0.251
***Lean mass***			
ASM/BMI	0.248±0.033	0.251±0.029	0.773
Arms lean mass (g)	3634.0±373.3	3258.0±404.9	**0.016**
Legs lean mass (g)	10487.0±1222.0	9665.0±1628.0	0.144
Trunk lean mass (g)	17260.0±522.5	16225.0±590.7	0.207
Thigh muscle CSA (mm^2^)	7998.8±1163.2	7637.1±1574.9	0.545
IMAT (mm^2^)	1265±52.6	1190±102.9	0.571
***Muscle function***			
Physical activity (hr/week)	1.08±1.38	2.19±3.10	0.243
OAK score (0-to-24)	15.5±4.3	16.4±4.8	0.578
***Circulating bone markers***			
cOC (ng/ml)	10.2±1.0	10.3±0.9	0.936
uOC (ng/ml)	3.7±0.6	4.5±0.9	0.509
Total ALP (U/L)	74.0±26.1	66.0±13.4	0.256
βCTx-I (ng/ml)	0.245±0.144	0.259±0.162	0.888
Total calcium (mg/dl)	9.3±0.3	9.4±0.5	0.427
PTH (pg/ml)	50.7±24.3	84.1±37.5	0.226
25-(OH)D (ng/ml)	36.4±12.8	33.7±14.3	0.900

Data are reported as mean ± SD. BMD, Bone Mineral Density; BMI, Body-Mass Index; ASMMI, Appendicular Skeletal Muscle Mass Index; CSA, Cross-sectional muscle area of the thigh; IMAT, Intermuscular fat of the thigh; cOC, carboxylated osteocalcin; uOC, uncarboxylated osteocalcin; ALP, alkaline phosphatase activity; PTH, Parathyroid hormone; 25-(OH)D, serum 25-hydroxyvitamin D; βCTx-I, beta-crosslinked C-terminal telopeptide of type I collagen. Bold values highlighted statistical significant values.

We further investigated about differences in cOC and uOC correlations between fractured and unfractured osteoporotic women (**Table 3**). The most striking difference between the two groups consists in the strong positive relationship between serum cOC and uOC levels in fractured women (r=0.852, p=0.0002), which was definitely abolished in unfractured women. In unfractured women, circulating cOC was confirmed to correlate with muscle mass parameters, such as legs lean mass, thigh CSA and IMAT. In fractured women, both cOC and uOC correlated with lean mass parameters, namely ASM/BMI and legs lean mass, with the OAK score-related risk of fall, and with bone markers, suggesting that the intense crosstalk among bone, and muscle mass and function mediated by cOC is more active in fractured osteoporotic women with respect to unfractured women.

**Table 3:** Correlations between cOC or uOC with the body composition parameters and bone markers in fractured and unfractured women.

OSTEOCALCIN	Fractured					Unfractured			
Parameters	cOC			uOC		cOC		uOC	
Pearson	r	P		r	P	r	P	r	P
**Muscle mass**									
ASM/BMI	0.433		0.139	0.716	**0.006**	0.100	0.712	-0.013	0.962
Legs lean mass (g)	0.661		**0.014**	0.499	0.084	0.595	**0.029**	-0.117	0.665
CSA (mm^2^)	0.378		0.282	0.050	0.891	0.569	**0.034**	0.190	0.515
IMAT (g)	-0.355		0.315	-0.453	0.189	0.640	**0.014**	0.095	0.747
**Muscle function**									
OAK score	0.645		**0.017**	0.591	**0.034**	0.170	0.530	-0.122	0.652
**Fat mass**									
BMI (kg/m^2^)	-0.200		0.521	-0.521	0.068	0.147	0.587	0.276	0.300
Total fat mass (g)	-0.221		0.468	-0.493	0.087	0.058	0.830	0.066	0.806
**Circulating bone markers**									
Total ALP (U/L)	0.786		**0.007**	0.794	**0.006**	0.494	0.214	0.665	0.072
βCTx-I (ng/ml)	0.855		**0.0002**	0.855	**0.0002**	0.063	0.817	0.678	**0.004**
25-(OH)D (ng/ml)	-0.689		**0.019**	-0.468	0.146	0.035	0.923	-0.494	0.147
uOC (ng/ml)	0.852		**0.0002**	–	–	-0.013	0.963	–	–

OC, carboxylated osteocalcin; uOC, uncarboxylated osteocalcin; fractured, osteoporotic women experiencing previous fragility fracture; unfractured, osteoporotic women without evidence of clinical or morphometric fractures; r, coefficient of correlation by Pearson test; P, statistical significance; ASM/BMI, appendicular skeletal muscle mass corrected for BMI; BMI, Body-Mass Index; CSA, Cross-sectional muscle area of the thigh; IMAT, Intermuscular fat of the thigh; ALP, alkaline phosphatase activity; βCTx-I, beta-crosslinked C-terminal telopeptide of type I collagen; 25-(OH)D, serum 25-hydroxyvitamin D. Bold values highlighted statistical significant values.

## Discussion

Osteoporosis and sarcopenia are serious health problems in postmenopausal women. In the present study, the interaction between bone and skeletal muscle was investigated in non-obese, non-diabetic, vitamin D-sufficient, older than 60 years postmenopausal osteoporotic women, free from anti-osteoporotic drugs and from any other treatment known to affect bone metabolism. The hypothesis that circulating carboxylated and/or uncarboxylated osteocalcin levels may mediate the bone-muscle interaction has been tested. Low muscle mass affected about a half of osteoporotic women, and data obtained by MRI suggested that elderly osteoporotic women with conserved lean mass may have, indeed, increased intramuscular fat infiltration, measured as IMAT. This finding is relevant because as IMAT increases, muscle quality, and possibly muscle function, decreases (43). Moreover, the frequently occurring low muscle mass at legs level and reduced thigh cross-sectional muscle area are known to be associated with reduced bone mineral densities, in line with a previous report (44), underscoring the relationship between bone and skeletal muscle in elderly osteoporotic women.

A consistent subset (40%) of osteoporotic postmenopausal women had a moderate-high increased risk of fall as evaluated by the automated system OAK. Interestingly, OAK score well correlated with muscle parameters, namely ASM/BMI and thigh CSA, supporting the hypothesis that a reduced muscle mass increases the risk of fall in osteoporotic women. Increased fat mass also emerged as a negative factor determining the risk of fall, though the role seems minor than that of age and ASM/BMI. Therefore, impaired muscle mass and function frequently occur in osteoporotic postmenopausal women and are related with BMD.

Experimental evidence suggest an intense crosstalk between bone and skeletal muscle through mechanic stimulation and hormones, including myokines, adipokines, and osteokines (15, 16). We focused attention on osteocalcin, whose endocrine function is emerging, though data are controversial (45). Gender differences in serum osteocalcin levels and their modulation have been reported (20), so to avoid this potential confounding bias, postmenopausal women have been analyzed in the present study. Moreover, osteoporotic women with diabetes and obesity were excluded, since the potential involvement of this hormone in the regulation of energy metabolism and endocrine pancreas function, at least in rodent models (46). Investigating both the carboxylated and uncarboxylated forms in a series of postmenopausal osteoporotic women, we found that both cOC and uOC levels positively correlated with serum βCTx-I levels, in line with what previously reported (47, 48), suggesting that OC is related with osteoclastic resorptive activity. We found that circulating cOC levels correlated with body composition, namely ASM/BMI, in particular with the trunk and legs lean masses, and with total fat mass in osteoporotic women. Of note, legs lean mass is mainly associated with circulating cOC levels, supporting the narrow relationship between skeletal muscle and bone-derived cOC in osteoporotic elder women. This finding represents a discrepancy with reports in mice, where uOC display endocrine action. The association we found between cOC and leg composition may be related to the muscular activity that, in humans, included the elderly, is prominent in the lower limbs. However, this finding should be confirmed and its physiological meaning might be investigated. Nonetheless, it is in line with the intervention study by Kyla Shea et al. (49) demonstrating that uOC was not cross-sectionally associated with appendicular lean mass or fat mass in older community-dwelling men or women, and that reduction of uOC levels following vitamin K supplementation affected neither lean nor fat mass over 3 years. Moreover, a positive correlation between circulating osteocalcin and lean mass has been reported in trained and untrained young women (50) and in middle-aged and elderly Chinese subjects (51). Moreover, exercise enhances osteocalcin serum levels in adult women (22), while osteocalcin circulating levels decrease during aging, when exercise capacity declines. By contrast, in community-dwelling middle-aged and elderly adults, Moriwaki et al. failed to detect any relationship between OC and muscle parameters (52).

Further, we focused the attention on the two subgroups of osteoporotic elder women who experienced at least one fracture and those who never suffered fractures, including screening for morphometric vertebral deformities. All fractures occurred at least 6 months before the enrollment in the study, when bone turnover markers return to baseline (53).

We failed in detecting any difference in body composition parameters, OAK score, as well as in circulating bone markers between the two groups. Nonetheless, fractured women were characterized by a positive relationship between circulating cOC and uOC, which was absent in unfractured women. Moreover, plasma cOC and uOC levels well correlated with total ALP and βCTx-I in fractured women, but not in unfractured women. These findings suggest that bone remodeling differ in osteoporotic fractured women compared with osteoporotic unfractured. In the present series of osteoporotic elder women, serum βCTx-I emerged as a determinant of circulating cOC, suggesting that osteoclasts-related resorptive activity, which is known to be associated with an increased risk of fractures (54, 55), may be involved in modulation of cOC levels, throughout its release from the matrix. Moreover, in fractured women, plasma cOC and uOC correlated with the lean mass parameters and OAK score, showing that relatively low circulating levels of cOC and uOC may be predictive of low legs lean mass and, hence, increased risk of fall in fractured osteoporotic women. Besides, unfractured women displayed a significant positive correlation of plasma cOC levels with IMAT and with legs lean mass, highlighting that relative high cOC levels may reflects conserved muscle mass though of reduced quality. This finding suggested that bone turnover rate may differ in fractured and unfractured osteoporosis and that, likely, differently influenced the release of the different forms of OC. The different correlations between cOC and uOC and the parameters of the musculoskeletal system, such as risk of fall and intramuscular fat infiltration, are consistent with a complexity in the modulation of the OC effect on skeletal muscle mass.

A limit of the present study is represented by the small sample size, that, though adherent to the power analysis calculation, is limited due to extensive exclusion criteria aimed to avoid a number of diseases and therapies known to independently affect bone mass and metabolism, as well as skeletal muscle mass and function. Second, study design prevents any inference about cause-effect relationship between plasma carboxylated osteocalcin and the different parameters analyzed. Third, vitamin K status, which is known to affect OC carboxylation, has not been evaluated in the present series due to unavailability of accurate tools such as high-performance liquid chromatography–tandem mass spectrometry for the assessment of vitamin K homologues, phylloquinone (vitamin K1) and menaquinones (MK-4 and MK-7). However, it is of note that measuring uncarboxylated vitamin K-dependent protein (i.e., osteocalcin) levels is considered the most accurate and convenient method for assessing tissue-specific vitamin K deficiency or insufficiency (56). Indeed, here failure in detecting association between plasma uOC levels and most of the parameters related with body composition, may suggest that vitamin K replenishment is not a major determinant of the potential relationship with muscle mass and function. Lastly, vitamin D was not routinely administered, though at the enrollment all women were checked for circulating 25-(OH)D levels and in all participants serum 25-(OH)D levels were above 20 ng/ml, a threshold considered consistent with a sufficient condition of vitamin D repletion (57).

Strengths of the present study are body composition analysis by DXA and risk of fall assessment by an automated system. DXA is the gold-standard technique in the analysis of body composition, providing assessment and quantification of fat mass, lean mass and bone mineral content, both in a single body region of interest and at whole-body level (35). The OAK system incorporates movement and balance sensors and accelerometers for the assessment of fall risk in a single examination. The diagnostic test accuracy of the OAK device has been recently investigated and the results showed good accuracy of OAK system in assessing risk of fall (discriminative power of AUC values above 80%) and the device also showed a sensitivity of 84% and a specificity of 67% (28). Diagnostic accuracy of OAK system was similar to the sensitivity levels obtained with other fall risk assessment, such as the Brief-BESTest. Moreover, data about skeletal muscle fat infiltration analyzed by lower leg MRI has been provided. Lastly, both circulating cOC and uOC levels were analyzed in the same sample, at variance with most clinical studies provided data about total OC and/or uOC.

In conclusion, data here presented supported the relationship between OC and skeletal muscle mass and function detected in mice, though in osteoporotic postmenopausal women cOC, but not uOC, emerges as mediator in the bone-muscle crosstalk. Though the release of cOC and uOC was similar, circulating levels may be differentially regulated in fractured and unfractured women, suggesting that bone metabolism differs in the two groups.

## Data Availability Statement 

The datasets presented in this study can be found in online repositories. The names of the repository and accession number can be found below: ZENODO http://doi.org/10.5281/zenodo.4558652.

## Ethics Statement

The studies involving human participants were reviewed and approved by Ospedale San Raffaele in Milan (ref.no.17/INT/2017). The patients/participants provided their written informed consent to participate in this study. All participants gave their signed informed consent to participation.

## Author Contributions 

SC, GL, and JV designed the study and prepared the first draft of the paper. SC is guarantor. JV, CM, and SC enrolled and clinically evaluated the patients. VS, MF, and CV contributed to the experimental work performing the assays. MF and SC were responsible for statistical analysis of the data. All authors agree to be accountable for the work and to ensure that any questions relating to the accuracy and integrity of the paper are investigated and properly resolved. All authors gave their approval and consent to publication; there is any restriction by government authorities. All authors contributed to the article and approved the submitted version.

## Funding

The study has been funded by the “Bando CARIPLO sulla ricerca biomedica legata alle malattie dell’invecchiamento 2018” for the project OSTMARK (no.2018-0458) and by Italian Ministry of Health.

## Conflict of Interest

The authors declare that the research was conducted in the absence of any commercial or financial relationships that could be construed as a potential conflict of interest.
